# Medical Society of the County of Erie

**Published:** 1911-06

**Authors:** Franklin C. Gram

**Affiliations:** Secretary


### SOCIETY PROCEEDINGS

### Medical Society of the County of Erie

REPORTED BY FRANKLIN C. GRAM, M.D., Secretary.

BY invitation of the Faculty of the Medical Department of the University of Buffalo, the regular meeting of the Medical Society of the County of Erie was held in Alumni Hall, 24 High Street, on Monday, April 10 1911, at 8.15 p. m.

President McClure presided.

The President stated that inasmuch as the minutes of the last regular meeting had been printed in the Journal, the reading of same would be omitted unless objections were offered. Hearing none, the minutes were declared adopted.

The Secretary read the minutes of the Council meetings of March 6 and April 3, 1911, which, upon motion, were received and filed.

Minutes of the Council meeting of April 3 contained three resolutions each of which were separately taken up.

The first resolution was unanimously adopted as follows:

*Resolved,* That the Board of Regents of the University of the State of New York be requested to recommend such change in the Medical Practice Act as would raise the requirements for securing the medical student certificate to include, in addition to the standard high school course, one year in college or its equivalent, embracing especially the following subjects: physics, chemistry (inorganic and qualitative analysis) and general biology.

*Resolved,* That the State Society recommend to the Board of Regents that an investigation be conducted in order to ascertain the number of internships available in the hospitals of the state with a view to recommending a fifth year in hospital for the completion of the medical curriculum.

The following is the second resolution referred to:

*Whereas,* During the past decade, the subject of public health has assumed a great importance, having to do with the conservation of human life, through the control of our potable water supplies, our food products, the introduction of infectious diseases into the country and from one state to another and in various other ways, and

*Whereas,* It has a great economic importance in that this conservation of human life necessarily adds to the wealth of the country, and

*Whereas,* The public health can be more effectively conserved and more efficiently directed from the seat of National Government, be it

*Resolved,* That the Congressional representatives of this district be requested to use their influence to secure the establishment of a National Health Bureau with a Commissioner of Public Health who shall have a seat in the President's cabinet.

After a short debate upon the subject, the foregoing resolution was adopted.

The third resolution was then read as follows:

*Whereas,* In the larger cities in the state of New York, nearly half of the births are attended by midwives, many of whom are wholly untrained for the responsible work which they assume, be it

*Resolved,* That the Erie County Medical Society favor the adoption of an educational standard similar in character to that which is now provided for trained nurses, and that it recommends that the State Medical Society take such action as may lead to the establishment of a standard, the requirement of adequate examinations, with universal registration of all midwives practising in this state.

Dr. F. Park Lewis, Chairman of the Committee on Legislation, made some explanatory remarks relative to this resolution.

Dr. Pryor said he thought that the resolution should be made much stronger and that arrangements should be made for the education and training of the midwives and, therefore, moved, as an amendment, that this society recommend that some provision be made, in the larger cities, for proper training and education of midwives.

Dr. Van Peyma said that the only trained midwives come from Europe, and that if you insist that the standard and education be raised, provision must be made for their training also.

The resolution, as amended by Dr. Pryor, was adopted. The entire minutes of the Council were then adopted, and the recommendations contained therein approved.

Dr. Wall, Chairman of the Committee on Membership submitted a list of candidates, each of whom was separately balloted upon and duly elected as follows: Edw. Villiaume, 398 South Park Avenue; George A. Stesel, 532 Winslow Avenue; J. H. Wild, 528 Elm Street; John Gurney Stowe, 743 Elmwood Avenue.

Dr. F. Park Lewis, read a letter from Dr. Green of Chicago, stating that Dr. McCormick, who represents the A. M. A., will be in this vicinity in the near future and asked that Dr. McCormick be given a hearty welcome to Buffalo. The exact date of Dr. McCormick's anticipated visit could not be stated.

Dr. Lewis moved that a committee be appointed from the County Society to take charge of the matter in conjunction with a similar committee from the Academy of Medicine.

Dr. Wende moved, as an amendment, that the subject matter be referred to the Council with power.

Dr. Lewis withdrew his motion and the amendment became an original motion and was adopted.

The business portion of the program being concluded, the following subjects were presented: A Few Ethical Suggestions, Dr. E. E. Haley; The Logic of Darwin with Regard to Man, Dr. F. M. O'Gorman; Some Clinical Cases, Dr. James W. Putnam; The Non-ventilation in our Public Schools, shown by lantern slides, Dr. H. R. Hopkins.

Each of the subjects produced a spirited discussion, and at the close of the discussion on Dr. Haley's paper, Dr. Wall moved that a committee of five be appointed to prepare rules governing the recommendations.

Dr. Pryor said his report on "Division of Fees," and which was adopted, provided for the appointment of such a committee, and suggested that Dr. Haley be appointed chairman.

Dr. Wall then withdrew his motion and the President said he would appoint such committee with Dr. Haley as chairman.

The members, at the close of the scientific program, adjourned to the Library where a collation was served.

